# Assessing the Contribution of the CFRP Strip of Bearing the Applied Load Using Near-Surface Mounted Strengthening Technique with Innovative High-Strength Self-Compacting Cementitious Adhesive (IHSSC-CA)

**DOI:** 10.3390/polym10010066

**Published:** 2018-01-11

**Authors:** Alyaa Mohammed, Nihad T. K. Al-Saadi, Riadh Al-Mahaidi

**Affiliations:** 1 Centre for Sustainable Infrastructure, Faculty of Science, Engineering and Technology, Swinburne University of Technology, P.O. Box 218, Melbourne, VIC 3122, Australia; 2 Department of Civil and Construction Engineering, Faculty of Science, Engineering and Technology, Swinburne University of Technology, P.O. Box 218, Melbourne, VIC 3122, Australia; nalsaadi@swin.edu.au (N.T.K.A.-S.); ralmahaidi@swin.edu.au (R.A.-M.)

**Keywords:** CFRP strips, near-surface mounted, cementitious adhesive, pull-out test, concrete

## Abstract

Efficient transfer of load between concrete substrate and fibre reinforced polymer (FRP) by the bonding agent is the key factor in any FRP strengthening system. An innovative high-strength self-compacting non-polymer cementitious adhesive (IHSSC-CA) was recently developed by the authors and has been used in a number of studies. Graphene oxide and cementitious materials are used to synthesise the new adhesive. The successful implementation of IHSSC-CA significantly increases carbon FRP (CFRP) strip utilization and the load-bearing capacity of the near-surface mounted (NSM) CFRP strengthening system. A number of tests were used to inspect the interfacial zone in the bonding area of NSM CFRP strips, including physical examination, pore structure analysis, and three-dimensional laser profilometery analysis. It was deduced from the physical inspection of NSM CFRP specimens made with IHSSC-CA that a smooth surface for load transfer was found in the CFRP strip without stress concentrations in some local regions. A smooth surface of the adhesive layer is very important for preventing localized brittle failure in the concrete. The pore structure analysis also confirmed that IHSSC-CA has better composite action between NSM CFRP strips and concrete substrate than other adhesives, resulting in the NSM CFRP specimens made with IHSSC-CA sustaining a greater load. Finally, the results of three-dimensional laser profilometery revealed a greater degree of roughness and less deformation on the surface of the CFRP strip when IHSSC-CA was used compared to other adhesives.

## 1. Introduction

Two-component epoxy resin organic adhesives are most frequently used for adhering FRP to reinforced concrete (RC) structural members, as the adhesive has a key role in any strengthening technique for RC structures. However, the use of these adhesives with strengthening techniques for RC structures has significant drawbacks because of the release of toxic fumes during curing, which may cause irritation and eczema to human skin. In addition, this type of organic adhesive is highly flammable [1]. Furthermore, at temperatures above 70 °C, polymer-based adhesive loses its properties [2]. Therefore, cement-based adhesives may be suitable alternative adhesive materials, since they have sufficient bonding properties [3,4]. However, polymer is still used as a major component in most cement-based adhesives. Recently, an innovative high-strength self-compacting non-polymer cementitious adhesive (IHSSC-CA) has been developed and used in the fabrication of many NSM-CFRP strengthened specimens. Specimens have been tested under different conditions: fire, static and fatigue loading. The results show significant enhancement of the performance of specimens compared with similar specimens fabricated with epoxy or other polymer-cement-based adhesive [5,6,7]. It is believed that the reason for these results is the sufficient load transfer in the bonding area, as IHSSC-CA maintains adequate load transfer on the surfaces of the CFRP strips, which results in increased load capacity and for other samples increased fatigue service life. The present research aims to shed light on why the use of IHSSC-CA results in a successful strengthening system. The research examines the bond zone in contact with the CFRP strip. Study of the bond zone is very important in providing a better understanding of the performance of strengthened specimens. It helps to explain both the weaknesses and strengths in order to overcome the defects and/or improve the strengths.

## 2. Experimental Work

Eighteen concrete prisms 75 × 75 × 250 mm^3^ were prepared for pull-out tests using different adhesives (IHSSC-CA, polymer cement and epoxy). Six NSM specimens were tested for each type of adhesive, three with strain gauges and three without strain gauges.

**Figure 1:**
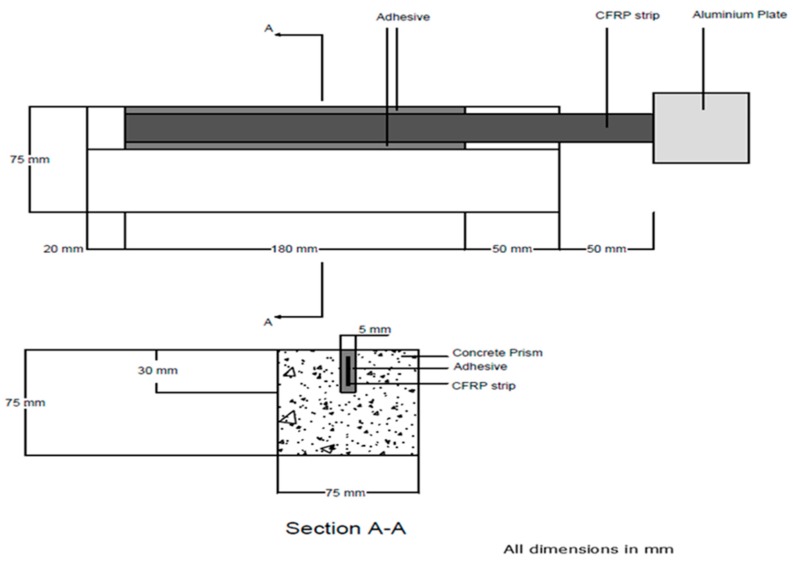
Specimens’ details.

A normal-strength concrete was used to cast the prisms. The mix design proportions by weight was 1:2.2:4.1 for cement: sand: gravel, according to ACI 211.1-91. The water/cement ratio was equal to 0.6 and the maximum size of gravel was 14 mm. The slump was about 50 mm consistent with ASTM C143-12. The concrete prisms were ready for making the grooves after 28 days moist curing. A table-mounted saw was used to cut a groove 5 × 30 mm^2^ suitable for placing a CFRP strip with a size of 1.4 × 20 mm^2^, according to ACI 440.2R-08. To enable load application, the CFRP strip was extended with at least 100 mm outside the concrete prism and attached with two aluminium plates with a dimension of 50 × 50 × 2 mm^3^ at the loaded-end of each CFRP strips. The specimens’ detail for pull-out test are shown in Figure 1. 

**Figure 2:**
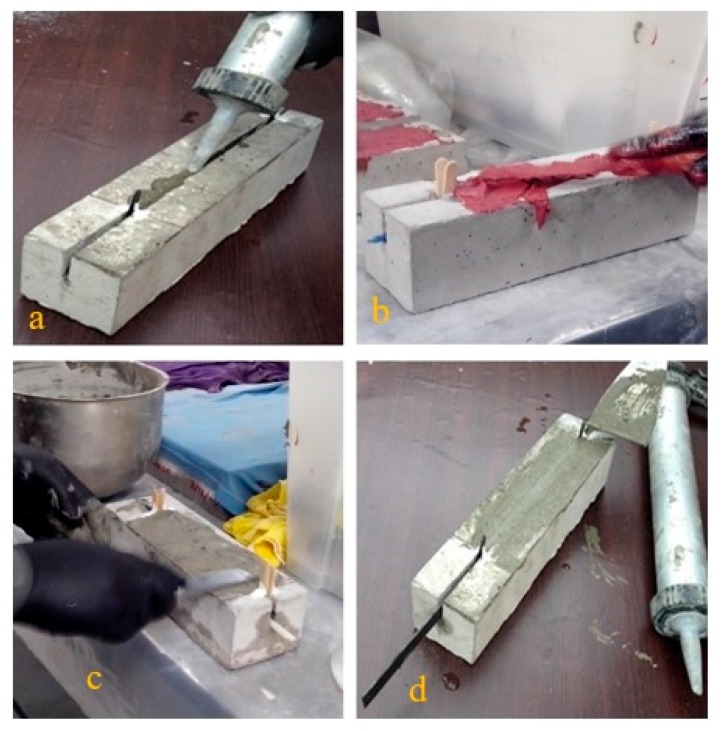
(**a**) IHSSC-CA specimen; (**b**) EA specimen; (**c**) PCA specimen; (**d**) Levelling of specimen’s surface.

Graphene oxide (GO), cementitious materials and additives were used to synthesise the IHSSC-CA and more details are found in Mohammed et al. [5]. The IHSSC-CA was prepared using an automatic mortar mixer according to the BS EN 196-1, BS EN 196-3, and BS EN 480-1 specifications. At the beginning, the dry materials were mixed for 2 min at high speed. Next, GO was added, followed by the mixing water and super-plasticizer. The CFRP laminate was cut to the required size and 50 mm was left unbonded at the loaded end to avoid specimen edge failure. Prior to applying the adhesive, the groove was wetted with water. Then, the groove was filled with IHSSC-CA using an injection gun. Next, the CFRP strip was inserted into the groove to the required depth. Finally, any excess adhesive was removed and the surface of the concrete prism was levelled. Owing to the high fluidity and self-consolidation of IHSSC-CA, ease of application was highly satisfactory. In addition, the self-consolidation property of IHSSC-CA avoids the formation of air voids inside the groove, which may affect the bond between the CFRP strip and the concrete substrate. Other adhesives used for comparison purposes were polymer-based cement adhesive (PCA and epoxy adhesive (EA) (BASF Australia Ltd., Melbourne, Australia) to fabricate the specimens for pull-out tests. The PCA used in this study is the same as that reported by Al-Abdwais et al. [8]. The specimens’ preparation are shown in Figure 2. 

The compressive strength and the tensile strength of the concrete, polymer and non-polymer cement-based adhesives at the age of testing were determined in accordance with ASTM C39-14a and ASTM C496-11, respectively, and the results are shown in Table 1. MBrace laminate adhesive (BASF Australia Ltd., Melbourne, Australia) was also used in this study and its properties, as specified by the manufacturer, are shown in Table 1. MBrace CFRP laminate 210/3300 (BASF Australia Ltd., Melbourne, Australia) was used in this work and its properties were determined based on tension tests in accordance with ASTM D 3039-14, as shown in Table 1.

## 3. Results and Discussion

### 3.1. Pull-Out Tests

**Figure 3:**
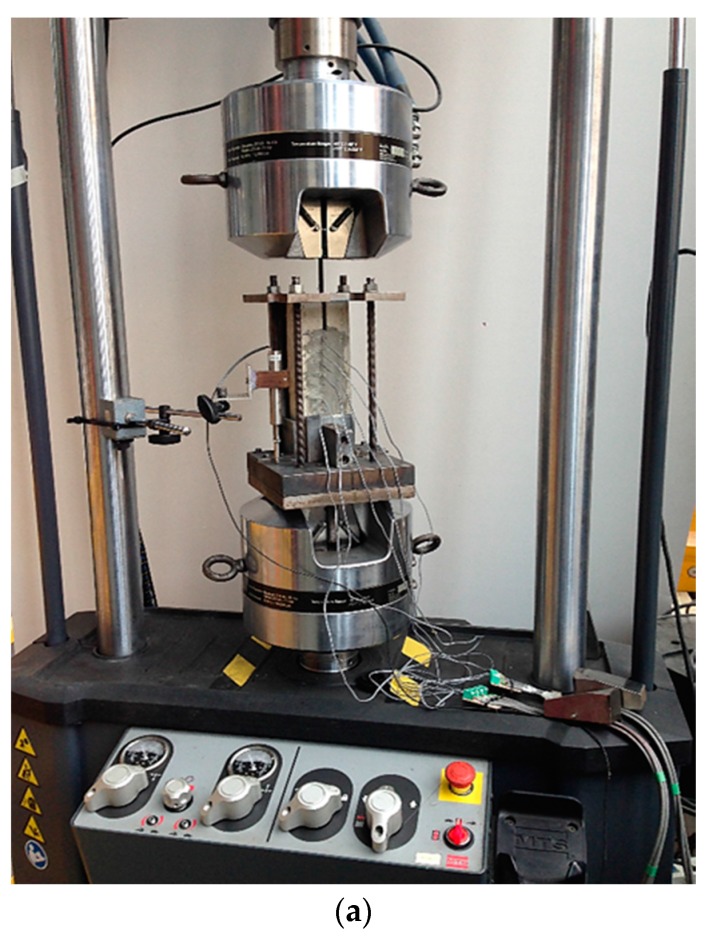

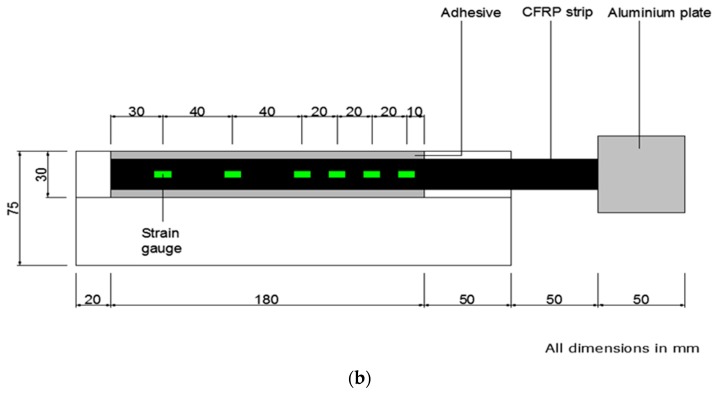
(**a**) Test set-up; (**b**) Strain gauges location.

The pull-out loads were applied at loading rate of 0.1 mm/min, vertical pulling load, by using a universal testing machine (250 kN capacity, MTS Systems Corporation, Eden Prairie, MN, USA), as shown in Figure 3. Each specimen was instrumented using one linear variable differential transformer (LVDT) (Mitutoyo America Corporation, Boulevard Aurora, IL, USA) for measuring the loaded-end slip (Figure 3). In order to study the bond response of NSM CFRP strips embedded in concrete substrate, the values of strain along the NSM CFRP strips were measured using six strain gauges (Figure 3). The strain gauges on CFRP strips were protected using M-Coat A poly coating and Dow Corning 3145 RTV adhesive (Dow Corning Corporation, USA).

The average value of three specimens was considered as the bond characteristic of the NSM pull-out specimens (Table 2). The results of pull-out tests showed comparable pull-out load for the specimens with IHSSC-CA of 34.5 KN as the epoxy specimens showed a load 41.0 KN. This result indicates the superior ability of IHSSC-CA to sustain the applied load and at the same time overcome the drawbacks of epoxy. The test results for the specimens with PCA showed pull-out load 22.2 KN, which is around 50% that of epoxy specimens.

NSM pull-out specimens with strain gauges show a reduction in strength compared to their counterpart specimens without strain gauges, as shown in Table 2. The reductions in strength were 34%, 22% and 17% for PCA-SG, IHSSC-CA-SG and EA-SG specimens, respectively. This may be because installing the strain gauges along the CFRP strips led to a reduction in the bonded length and finally caused weakening of the NSM bonding system, which led to failure at low pull-out forces.

**Figure 4:**
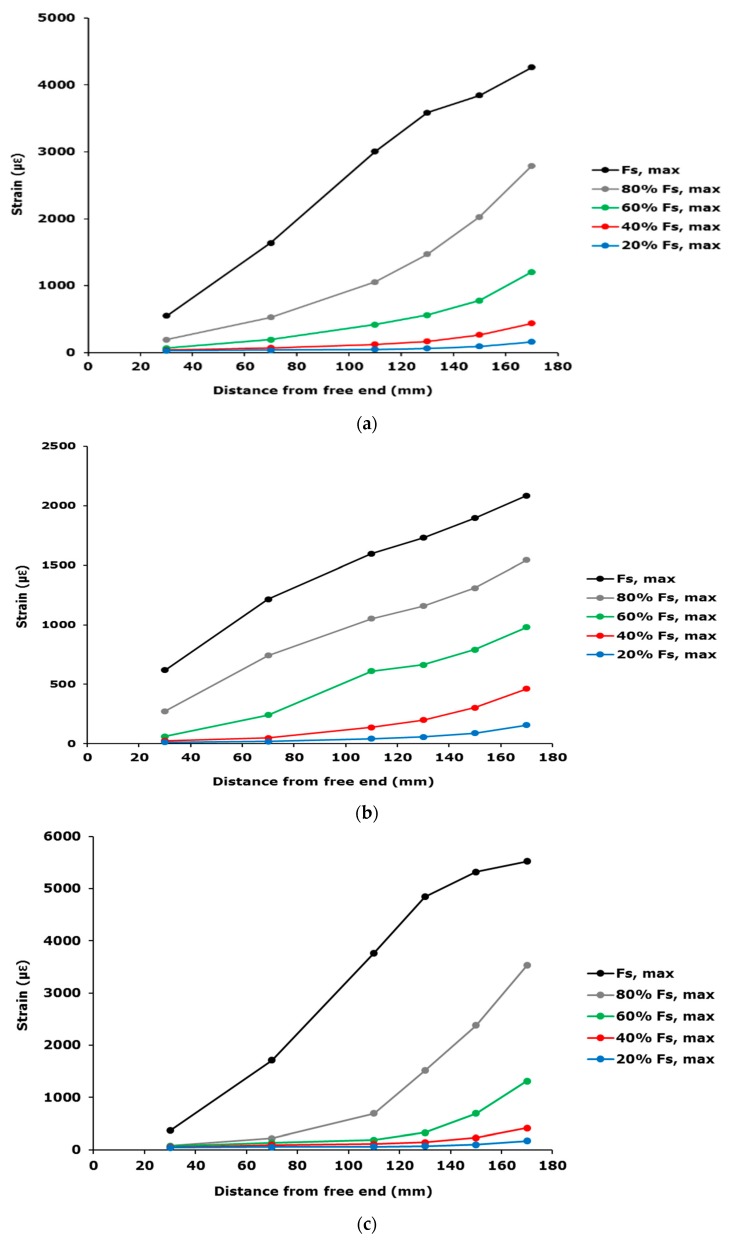
(**a**) IHSSC-CA–SG; (**b**) PCA–SG; (**c**) EA-SG.

The strain distributions in the NSM CFRP strips are shown in Figure 4. The average values of three specimens are displayed in Figure 4. The results showed comparable strain values for the specimens with innovative cement adhesive (IHSSC-CA-SG) compare to that of epoxy specimens (EA-SG). At the same time, the results for the specimens with polymer cement adhesive (PCA-SG) showed non-comparable strain readings compare to epoxy specimens (EA-SG). This may be because of the low mechanical strengths of PCA. 

**Figure 5:**
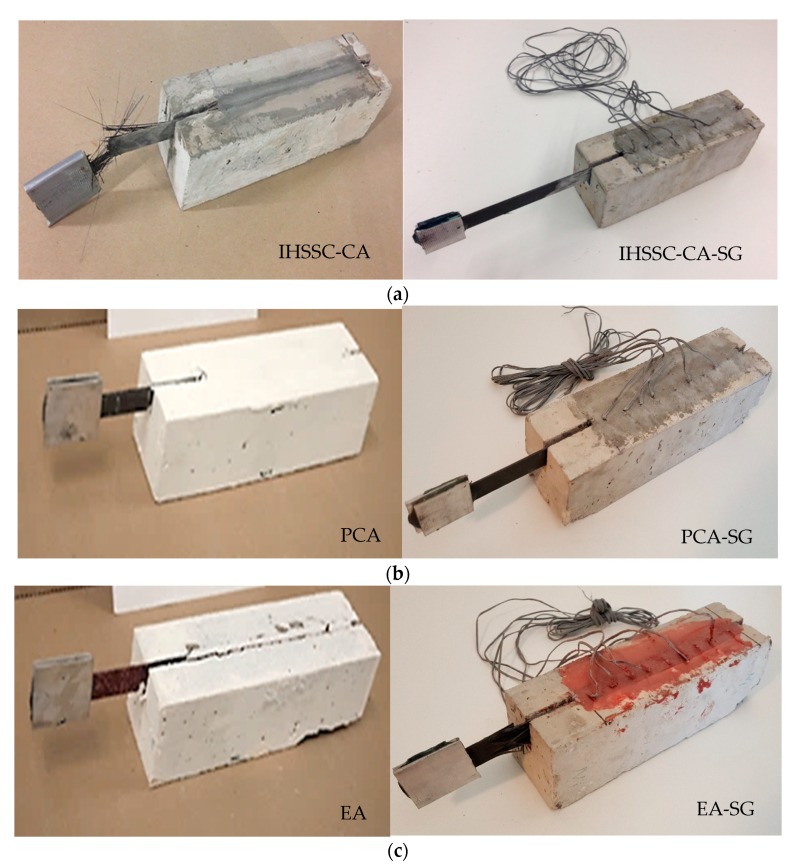
(**a**) IHSSC-CA and IHSSC-CA-SG specimens; (**b**) PCA and PCA-SG specimens; (**c**) EA and EA-SG specimens.

The IHSSC-CA specimens failed by rupture of CFRP strips and no cracks were noticed on the surfaces of the concrete or along the bonded length. This mode of failure occurs as a result of the efficient confinement between concrete and the IHSSC-CA and CFRP strips. When the loaded-end slip at the peak pull-out strength of NSM specimens is greater than the maximum allowable elastic elongation of the CFRP strips and above the peak pull-out strength, the carbon fibre of CFRP strips fractures gradually during the progress of the test near the loaded end until failure, as shown in Figure 5a.

The IHSSC-CA-SG specimens failed by pull-out of the CFRP strip. This type of failure occurs at the NSM CFRP strip and cement-based adhesive (IHSSC-CA) interface. Installing the strain gauges along the CFRP strips led to reduced bonded length and finally caused weakening of the NSM bonding system. This led to failure by pull-out of the CFRP strip from the cement-based adhesive (IHSSC-CA), as shown in Figure 5a.

The PCA and PCA-SG specimens failed by pull-out of CFRP strips and no cracks were observed on the concrete surfaces (Figure 5b). This type of failure occurs due to slipping at the NSM CFRP strip–adhesive interface because of the weak bond between the adhesive and the CFRP strip, which may be due to the low tensile strength of the polymer cement-based adhesive.

The EA specimens failed by debonding of CFRP strips and cracks were noticed on the surfaces of the concrete and along the bonded length (Figure 5c). This mode of failure happens due to the high applied shear stress with cracking spreading in the concrete next to the CFRP strips.

The EA-SG specimens failed by rupture of CFRP strips and few cracks were noticed along the bonded length with no cracks on the surfaces of the concrete. Installing the strain gauges along the CFRP strips led reduction of the bonded length and finally caused reduction in the applied shear stress between CFRP strips and concrete substrate. This type of failure happens for the reason that the loaded-end slip at the peak pull-out strength of NSM specimens is greater than the maximum allowable elastic elongation of the CFRP strips. Above the peak pull-out strength, the carbon fibre of the CFRP strips fractures gradually during the progress of the test outside the bonded length until failure, as shown in Figure 5c.

### 3.2. Physical Inspection

**Figure 6:**
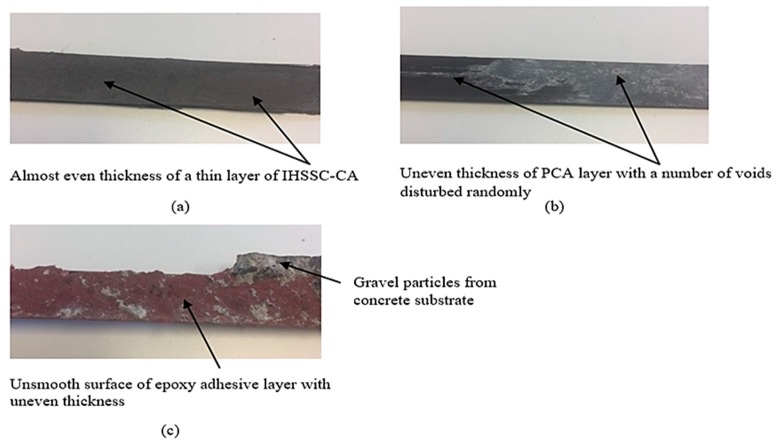
Images of bond area after pull-out testing, (**a**) IHSSC-CA specimen; (**b**) PCA specimen; (**c**) EA specimen.

Valuable information to investigate reasons beyond significant bond strength can be derivative from physical examining of bond area after the pull-out tests. Figure 6 shows images of the physical inspection of the CFRP strips used in the pull-out tests for sample with IHSSC-CA, PCA and EA. It can be seen from Figure 6a that IHSSC-CA effectively encapsulates the CFRP strip along the bond length where the CFRP strip completely covered by IHSSC-CA. It can be concluded that, due to the liquid-like nature of IHSSC-CA, it fully and equally coats CFRP strip and maintains a sufficient surface area of adhesive in the bond area. In addition, the self-compacting characteristic of IHSSC-CA works effectively to eliminate the creation of air voids during the pouring of adhesive into the groove. These physical properties of high fluidity and zero compaction have been shown to be important for the development of considerable bond strength. Figure 6b shows a sample with polymer based-cement adhesive (PCA), and it can be seen that there are many defects in the adhesive layer along the surface of the groove. Theses defects may be created due to difficulty to consolidate the adhesive layer during the application process. As a result, air voids were created and caused non-uniformed settlement of the adhesive layer. It can be deduced that, due to the addition of polymer in the cementitious mixture, the fluidity of the adhesive and pot life were reduced and this reduced the ease of application of the adhesive layer uniformly and evenly. Furthermore, Figure 6c showing the CFRP strip with epoxy (EA), it can be seen that the use of high viscosity adhesive such as epoxy causes difficulty in maintaining a uniform thin layer of the adhesive. It is clear that a uniform thin layer of adhesive was not achieved due to the high viscosity of the epoxy and low pot life, which make it difficult to obtain an even distribution of the adhesive layer on the attached surfaces. As a result, this uneven layer of epoxy created a weak bond zone with many defects. In addition, it can be concluded that the areas on the attached surfaces with more adhesive create regions for stress concentration which subsequently caused sudden failure of the tested samples. Many parts of the concrete substrate with gravel particles attached on the surface of the CFRP strip were observed, as shown in Figure 6c. This shows that a localized stress concentration occurred in these regions and caused a brittle failure of the concrete substrate. 

### 3.3. Pore Structure Analyses

**Figure 7:**
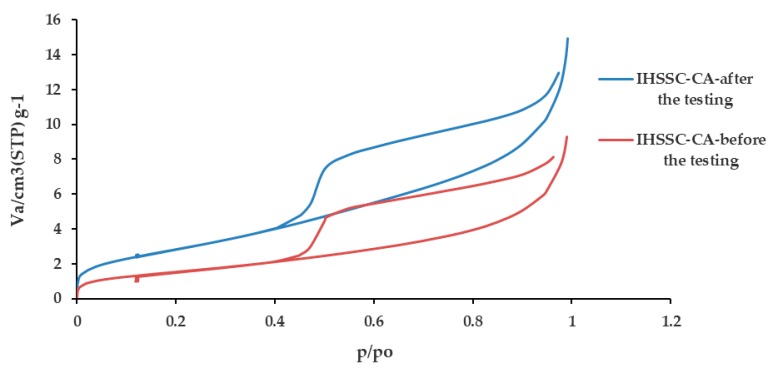
Nitrogen adsorption isotherms for IHSSC-CA.

**Figure 8:**
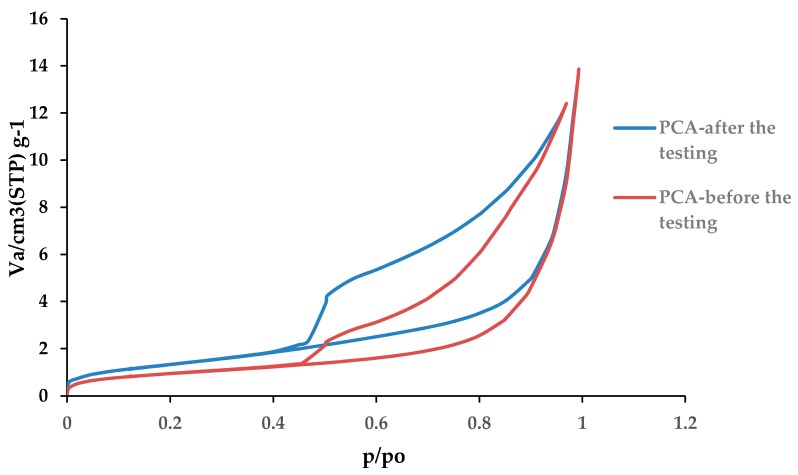
Nitrogen adsorption isotherms for PCA.

Burnauer-Emmett-Teller (BET) isotherm analysis is frequently used to study the adsorption properties of many types of materials [9]. In the present research, nitrogen adsorption isotherms were used to investigate the pore structure of the adhesives before and after pull-out testing. The results can be used to infer the contribution of adhesives to the NSM CFRP strengthening technique in resisting applied load. The adhesives were IHSSC-CA and polymer cement-based adhesive (PCA). It is not appropriate to conduct this test for epoxy adhesive (EA) because epoxy has very low permeability, which make it unsuitable for nitrogen adsorption testing. A Bell nitrogen adsorption machine was used for the tests at an adsorption temperature of 77 K. Figure 7 shows the nitrogen adsorption isotherms for unloaded IHSSC-CA and after applying the pull-out test. It can be seen from Figure 7 that IHSSC-CA shows greater adsorption after applying pull-out test than the unloaded IHSSC-CA, with more pronounced hysteresis loops. This observation indicates that the IHSSC-CA of the NSM specimens experienced more damage in the microstructure during loading. This reflects great contribution of IHSSC-CA to transferring the applied loads. Figure 8 shows the nitrogen adsorption isotherms for unloaded PCA and after applying the pull-out test. In contrast, the adsorption isothermal curves of PCA show a slight change in adsorption of this adhesive, which means little damage of microstructure during the loading. Therefore, it can be deduced that PCA makes a lesser contribution to transferring the applied load. 

### 3.4. Three-Dimensional Laser Profilometery Analyses

**Figure 9:**
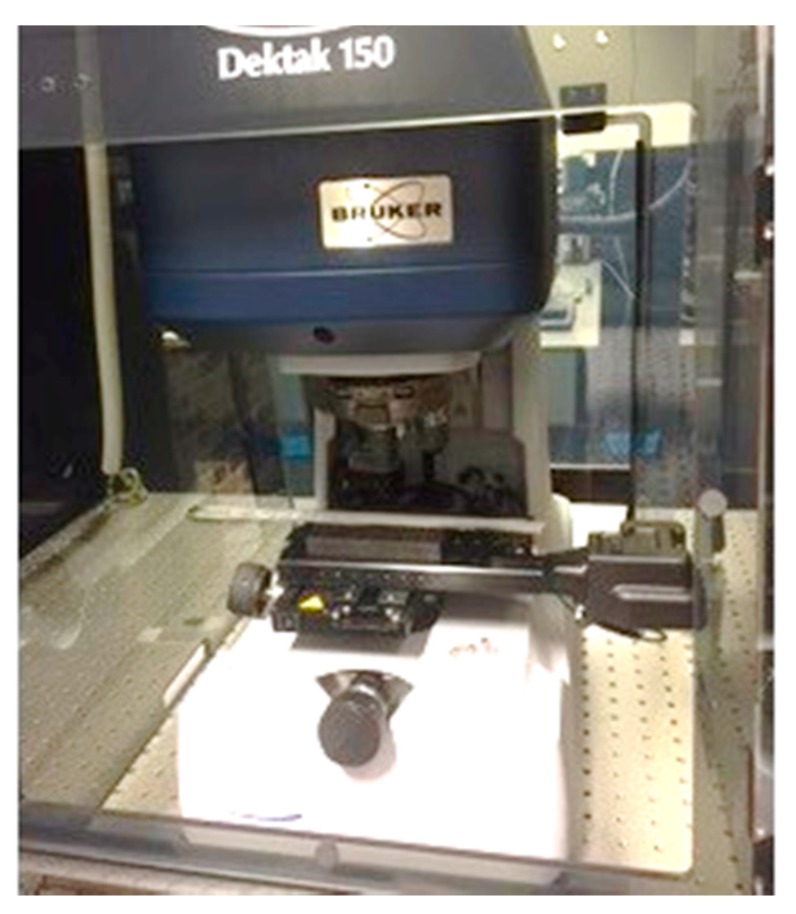
Laser profilometery of CFRP surface after failure analysis.

3-Dimentional (3-D) laser interferometry was used to investigate the topography (roughness) of the CFRP strip prior and after pull-out testing. It is of a great importance to measure and characterise the topography of the CFRP strips in order to evaluate the adequacy and efficiency of the bonding system. Samples of the CFRP strips were collected from similar positions around the mid-span of the CFRP strips of the tested specimens; the length of samples tested was about 18 cm. The samples were cleaned thoroughly before placing them in the profilemetry machine. 3-D micrographs of the CFRP strips were taken using a Bruker vertical scanning interferometer (Nanotech photomap 3D), as shown in Figure 9. 

**Figure 10:**
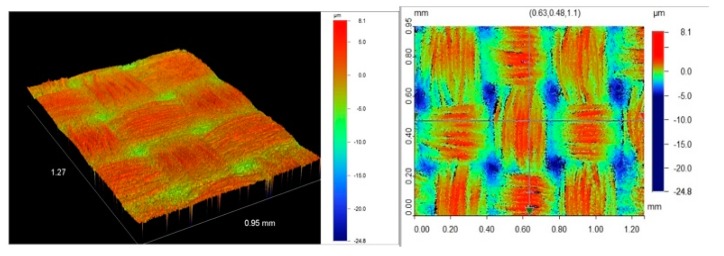
3-D and 2-D images of topographic surface of plain CFRP strip.

The surface roughness parameters were included *R*_a_ which is micro-surface roughness, in other words it describes average roughness. The other parameters observed in the test were *R*_q_, root mean average; *R*_t_, the peak-to-valley difference; and *R*_v_, the maximum profile valley depth. Veeco 3-D software based on ANSI B46.1 was adopted for calculating values of the surface roughness parameters. The same software was used for constructing the 3-D analyses of the captured images. The analyses results were then used to explain the utilization of the CFRP strips [10,11]. Some researchers refered to the importance of the nature of the CFRP strip-adhesive bond and described the bond as an effective factor in both of decreasing and/or increasing the overall strength and durability of strengthening systems [12,13]. More specifically, the first layer of adhesive that contacts the CFRP strip plays an important role in developing strong bond. Subsequently, a dense and uniform adhesive layer can effectively improve development of strength in strengthening systems [13]. However, a bond area with an adhesive layer that containing many defects will increasingly degrade and lose load capacity [12]. Therefore, the roughness or surface texture in bond zone is a result of the mechanical response of the solid material (CFRP strip), which can be vesuilized in morphological differences produced by the corresponding failure [14]. Figure 10 shows 3-D and 2-D images of a plain CFRP strip, and the 2-D woven textile of the strip is cleary seen. Table 3 shows the roughness parameters of the plain CFRP strip and the CFRP strips with different adhesives after application of the load. 

**Figure 11:**
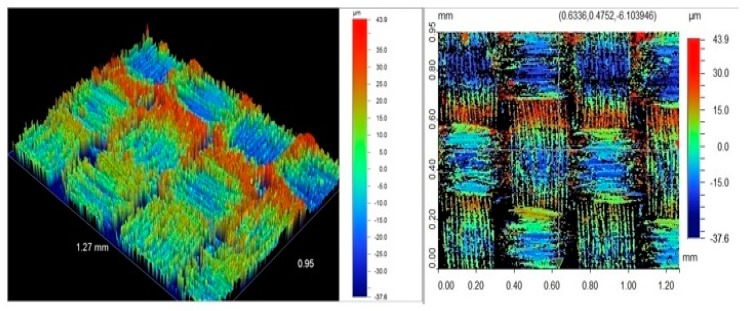
3-D and 2-D images of topographic surface of CFRP strip with IHSSC-CA.

**Figure 12:**
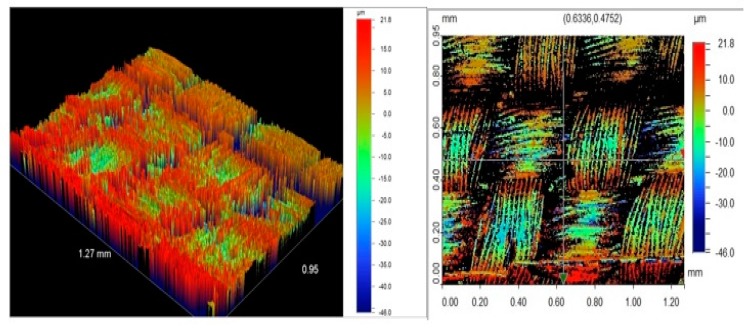
3-D and 2-D images of topographic surface of CFRP strip with PCA.

**Figure 13:**
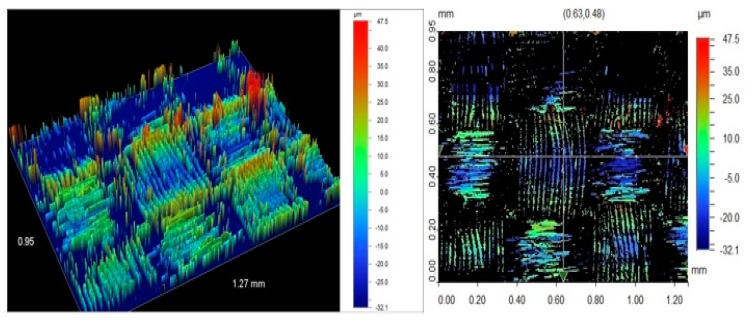
3-D and 2-D images of topographic surface of CFRP strip with EA.

Examining of the surface of the plain CFRP strip was used as a reference for comparison with surface differences after application of the load. The analysis revealed a clear difference in the surface roughness characteristics of the CFRP strips after the application of load. Figure 11, Figure 12 and Figure 13 show the CFRP strip after applying the pull-out load for the specimens strengthened with IHSSC-CA, PCA and EA, respectively. It can be deduced from these figures and from roughness values shown in Table 3 that the roughness of the CFRP was greater when IHSSC-CA was used compare to other adhesives. This can be attributed to formation of a uniform adhesive layer, which was able to successfully distribute the applied stress uniformaly and simulate more threads of the CFRP strip to resist the applied load. Another important factor that can be considered is the better interlocking of IHSSC-CA with the threads of CFRP strip due to high fluidity of IHSSC-CA. As a result, the *R*_t_ (81.5 µm) and *R*_v_ (74.5 µm) values were higher than those for the other adhesives. This is also shown in the surface of the CFRP strip in Figure 11, where there is a uniform deformation of the woven threads of the CFRP strip, which shows that almost every individual thread of the CFRP strip was affected by the applied load. On the other hand, the deformation in the surface of the CFRP strip with PCA and EA was severe and not uniform. This non-uniform deformation in the surface of the CFRP strip may be caused by uneven distribution of the adheive (PCA and EA) on the surface of the CFRP strip. It may also be caused by the weak interlocking effect between the PCA and EA adhesives and the threads of the CFRP strip because of the high viscosity of the polymer which is a base component of both PCA and EA.

## 4. Conclusions

Several tests were conducted on CFRP strips after direct pull-out tests to investigate the effects of bond system properties. All the results from the investigation show high agreement with those of the pull-out tests. Physical investigation of the CFRP strips showed the effectiveness of obtaining a thin uniform adhesive layer that is able to transfer the applied loads efficiently by using IHSSC-CA and prevent the formation of stress concentration regions such as those formed in EA and PCA. In addition, pore structure analysis showed considrable change in the pore structure of IHSSC-CA after pull-out loading which showed a good response to the applied load. In contrast, PCA showed lower response to the applied load representive by low modification in the pore structure of this adhesive. Finally, the results of 3-D laser profilometery showed a high degree of roughness and less deformation in the surface of the CFRP strip when IHSSC-CA was used compared to EA and PCA adhesives. It can be concluded that IHSSC-CA can be used as an alternative to epoxy adhesive, since it provides high strength and it can withstand harsh environments. 

## Figures and Tables

**Table 1:** Material properties.

Material	Compressive Strength (MPa)	Tensile Strength (MPa)	Modulus of Elasticity (GPa)
Concrete	41.1	4.2	38.4
Innovative non-polymer cement-based adhesive (IHSSC-CA)	116.7	18.6	45.7
Polymer cement-based adhesive	61.2	6.6	30.6
MBrace laminate adhesive	60	32	10
MBrace CFRP laminate	_	3697	212

**Table 2:** Pull-out test results.

Specimens ID	Maximum Pull-Out Force (kN)	Maximum Bond Strength * (MPa)	Failure Mode
IHSSC-CA	34.5	4.8	Rupture of CFRP strip
IHSSC-CA-SG	27.2	3.7	Pull-out of CFRP strip
PCA	22.2	3.0	Pull-out of CFRP strip
PCA-SG	14.7	2.0	Pull-out of CFRP strip
EA	41.0	5.7	Debonding of CFRP strip
EA-SG	34.0	4.7	Rupture of CFRP strip

* Maximum bond strength calculated by dividing maximum pull-out force by contact area between adhesive material and CFRP strips.

**Table 3:** Roughness parameters (µm) of the plain CFRP strip and CFRP strips with different adhesives after application of load.

Specimen	*R*_a_	*R*_q_	*R*_t_	*R*_v_
Plain CFRP strip	1.3	1.7	44.5	38.0
CFRP strip with IHSSC-CA	11.4	13.6	81.5	74.5
CFRP strip with PCA	8.6	10.7	67.4	65.4
CFRP strip with EA	9.9	12.1	77.6	61.8

## References

[1] Täljsten B., Blanksvärd T. (2007). Mineral-based bonding of carbon FRP to strengthen concrete structures. J. Compos. Constr..

[2] Gamage J.C.P.H., Al-Mahaidi R., Wong M.B. (2006). Bond characteristics of CFRP plated concrete members under elevated temperatures. Compos. Struct..

[3] Hashemi S., Al-Mahaidi R. (2012). Experimental and finite element analysis of flexural behavior of FRP-strengthened RC beams using cement-based adhesives. Constr. Build. Mater..

[4] Al-Saadi N.T.K., Al-Mahaidi R., Abdouka K. (2016). Bond behaviour between NSM CFRP strips and concrete substrate using single-lap shear testing with cement-based adhesives. Aust. J. Struct. Eng..

[5] Mohammed A., Al-Saadi N.T.K., Al-Mahaidi R. (2016). Bond behaviour between NSM CFRP strips and concrete at high temperature using innovative high-strength self-compacting cementitious adhesive (IHSSC-CA) made with graphene oxide. Constr. Build. Mater..

[6] Al-Saadi N.T.K., Mohammed A., Al-Mahaidi R. (2017). Performance of RC beams rehabilitated with NSM CFRP strips using innovative high-strength self-compacting cementitious adhesive (IHSSC-CA) made with graphene oxide. Compos. Struct..

[7] Al-Saadi N.T.K., Mohammed A., Al-Mahaidi R. (2017). Fatigue performance of NSM CFRP strips embedded in concrete using innovative high-strength self-compacting cementitious adhesive (IHSSC-CA) made with graphene oxide. Compos. Struct..

[8] Al-Abdwais A., Al-Mahaidi R., Abdouka K. Modified cement-based adhesive for near-surface mounted CFRP strengthening system. Proceedings of the 4th Asia-Pacific Conference on FRP in Structures (APFIS 2013).

[9] De Belie N., Kratky J., Van Vlierberghe S. (2010). Influence of pozzolans and slag on the microstructure of partially carbonated cement paste by means of water vapour and nitrogen sorption experiments and BET calculations. Cem. Concr. Res..

[10] Santos P.M., Julio E.N. (2007). Correlation between concrete-to-concrete bond strength and the roughness of the substrate surface. Constr. Build. Mater..

[11] Santos P.M., Júlio E.N. (2013). A state-of-the-art review on roughness quantification methods for concrete surfaces. Constr. Build. Mater..

[12] Hoła J., Sadowski Ł., Reiner J., Stach S. (2015). Usefulness of 3D surface roughness parameters for nondestructive evaluation of pull-off adhesion of concrete layers. Constr. Build. Mater..

[13] Erdem S., Blankson M.A. (2013). Fractal–fracture analysis and characterization of impact-fractured surfaces in different types of concrete using digital image analysis and 3D nanomap laser profilometery. Constr. Build. Mater..

[14] Ficker T., Martišek D., Jennings H.M. (2010). Roughness of fracture surfaces and compressive strength of hydrated cement pastes. Cem. Concr. Res..

